# Late-onset recurrent lupus nephritis in a kidney transplant recipient 

**DOI:** 10.5414/CNCS111897

**Published:** 2026-05-11

**Authors:** Jessica K. Cobb, Paul Endres, Prashamsa Shenoy, Edward J. Filippone, Maitreyee Gupta, Rakesh Gulati

**Affiliations:** 1 Department of Transplant Nephrology, and; 2 Department of Internal Medicine, Thomas Jefferson University Hospital, Philadelphia, PA, USA

**Keywords:** kidney transplant, nephrology, lupus

## Abstract

Long-term kidney allograft survival continues to be a challenge, despite improvements in the short term. Recurrence of glomerular disease is a significant cause of allograft failure. We report a case of graft dysfunction 27 years post transplant attributed to biopsy-proven recurrent class IV lupus nephritis. Following targeted medication adjustments aimed at treating lupus nephritis, the patient achieved complete remission within 6 months, which has been sustained for 4 years. Although recurrent lupus nephritis is reported to occur up to 16 years after transplantation, it is uncommon, and represents a potentially treatable cause of graft failure. This report underscores the critical need for heightened awareness and prompt management of recurrent lupus nephritis regardless of the time post transplant to enhance long-term allograft survival. Furthermore, our case highlights that positive allograft staining in peritubular capillaries for C4d may result from recurrent immune-complex disease as opposed to antibody-mediated rejection.

## Introduction 

The reported recurrence rate of lupus nephritis in renal allografts varies widely from 1 to 54% [1, 2, 3, 4, 5, 6, 7]. The documented timeline of recurrence also has a wide range, occurring as early as days post transplant to as late as 16 years. Many cases of recurrent lupus nephritis (rLN) tend to be mild and manageable with immunosuppressive therapy, but allograft survival may be shortened. We present a case of clinically significant, biopsy-proven class IV rLN that was diagnosed 27 years after transplantation. This case highlights the necessity of considering rLN as a cause of kidney allograft dysfunction regardless of the duration post transplant and the potential for positive outcomes even decades later. 

## Case report 

A Latino man was diagnosed in 1988 with systemic lupus erythematosus (SLE) at the age of 21. Subsequently, he developed end-stage kidney disease (ESKD) from biopsy proven class IV lupus nephritis and received a preemptive, living-related kidney transplantation in 1992 from his mother. He was HLA A(1) A2, HLA A(2) A28, HLA B(1) B40, DRB(1) DR4, ABO O. His mother was a 3-antigen mismatch with a CMV moderate risk profile. Chronic immunosuppression included azathioprine 100 mg daily and prednisone 10 mg every other day for 25 years, and he reported complete compliance. 

In the summer 2020, 27 years after transplantation, the patient presented for a routine checkup and allowed labs to be drawn. The serum creatinine (Cr) was 1.51 mg/dL (reference range 0.76 – 1.27 mg/dL), compared to 1.43 mg/dL in the summer of 2015. The urine protein-to-creatinine ratio (Prot:Cr ratio) was 500 mg/g (reference range 0 – 200 mg/g Cr) without previous values available. The patient denied symptoms of lupus activity including fever, rash, arthralgias, chest pain, cough, shortness of breath, rhinorrhea, epistaxis, myalgia, hematuria, ankle swelling, hair loss, or psychological issues. C-reactive protein (CRP) was 17.2 mg/L (reference range 0 – 10 mg/L), erythrocyte sedimentation rate (ESR) was 104 mm/h (reference range 0 – 30 mm/h), double-stranded (ds) DNA Ab was 16 IU/mL (reference range ≥ 10 positive), C3 was 73 mg/dL (reference range 82 – 185 mg/dL), C4 was 5 mg/dL (reference range 15 – 53 mg/dL), IgG cardiolipin antibody was 75 U (reference range 21 – 80 U low medium positive), IgM cardiolipin antibody was 116 U (reference range > 80 U high positive), IgG B2 glycoprotein antibody was > 150 U (reference range ≤ 20 SAU), and IgM B2 glycoprotein antibody was > 150 U (reference range ≤ 20 SAU). 

The combination of elevated ESR, CRP, positive dsDNA titer, and depressed complement (C3 and C4) suggested a lupus flare despite the absence of symptoms and signs of active SLE. A biopsy revealed lupus nephritis, class IV with mild activity (5/24) and mild chronicity (3/12). One of 14 glomeruli was globally sclerosed. On immunofluorescence microscopy, IgG, IgA, IgM, C1q, κ, and λ were diffusely positive (all 3+) in the glomerular capillary walls and mesangium. Peritubular capillary staining for C4d was 2+. Electron microcopy revealed prominent subendothelial and mesangial electron-dense deposits. Electron-dense deposits were also found adjacent to the peritubular capillary basement membrane. There was segmental foot process effacement accompanied by peripheral aggregation of intracellular actin microfilaments. The bodies of the podocytes were vacuolated, and segmental villous transformation of the cytoplasm was noted. The glomerular basement membranes were intact throughout without thickening of the lamina dense. Reduplication of the basement membrane was not present. Endothelial cells were swollen with loss of their fenestrations. Tubulo-reticular inclusions were evident in the cytosol of the endothelial cells. 

**Figure 1. Figure1:**
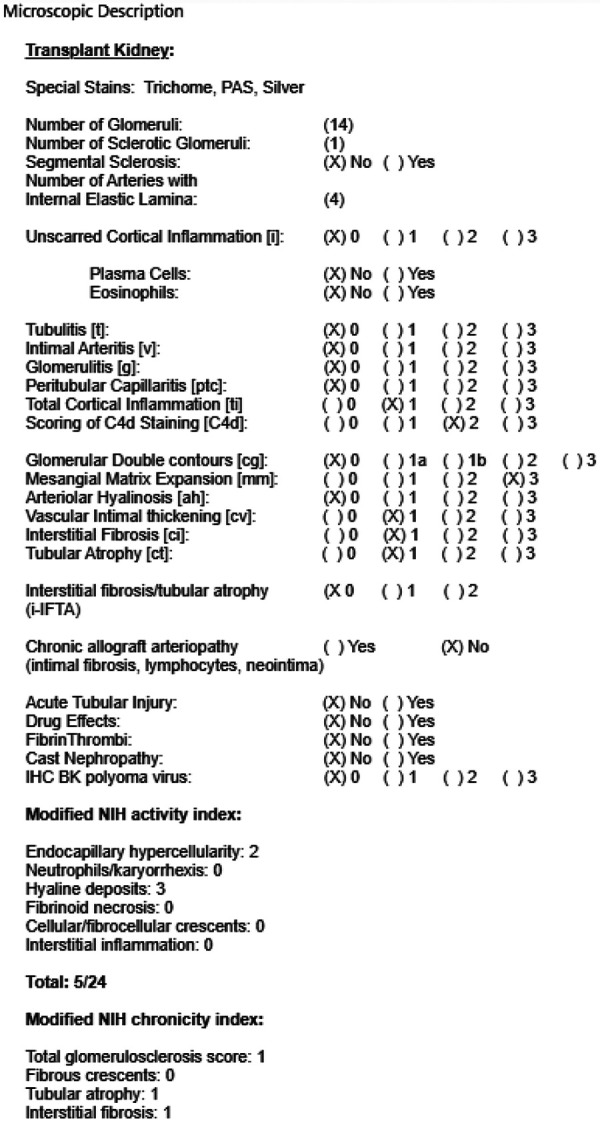
Renal biopsy microscopic description.

Given the biopsy-proven diagnosis of class IV lupus nephritis, immunosuppression was switched from azathioprine to mycophenolate sodium (720 mg twice daily), and prednisone was continued at 10 mg every other day. Subsequently, kidney function slowly improved. After 3 months, Cr was 1.48 mg/dL, and after 6 months it was 1.22 mg/dL. The Prot:Cr ratio decreased to 143 mg/g at 3 months and 128 mg/g at 6 months. He remains asymptomatic from the lupus standpoint 4 years after the biopsy, 32 years after transplantation, with stable kidney function (Cr 1.26 mg/dL and Prot:Cr ratio 124 mg/g) (Table 1)[Fig Figure1]. 

## Discussion 

In the United States, lupus nephritis accounts for 1.9% of cases of ESKD, with 5 – 20% of individuals with lupus nephritis progressing to ESKD [8, 9, 10, 11]. Kidney transplantation is the preferred modality for ESKD caused by lupus nephritis, and is preferred over long-term dialysis [12]. Transplant outcomes show comparable patient and graft survival rates to other causes of ESKD [13, 14, 15]. 

rLN refers to the reappearance of lupus nephritis in the renal allograft in a patient with SLE after kidney transplantation and is characterized by immune complex-mediated glomerulonephritis which can lead to graft dysfunction and potentially graft loss. Risk factors for rLN include high anti-dsDNA antibody titers and low compliment levels at time of transplantation [16]. 

The diagnosis of rLN entails clinical, laboratory, and histopathological evaluations. Suggestive laboratory tests include increased proteinuria, increased serum creatinine, and/or change in lupus serologies [12]. Kidney biopsy is the gold standard for confirming the diagnosis. Historically, rLN recurrence post transplant was considered rare, estimated at 1 – 4%. However, recent studies indicate a higher incidence with considerable variability (Table 2). For example, a study by Yu et al. [1] evaluating transplant biopsies from 23 Chinese kidney transplant recipients with ESKD from lupus nephritis found that 26% (6 out of 23 patients) developed rLN, with a mean time to diagnosis 5.1 ± 4.9 years post transplantation (range: 1.8 months to 11.6 years). 

In a larger cross-sectional biopsy study, Norby et al. [2], observed a recurrence rate at 54% (22 patients out of 41) with a mean time to biopsy of 8 years post transplant. The majority of these biopsies were performed for surveillance, with only 6 being conducted due to clinical indications [2]. 

Moroni et al. [3] demonstrated a 8.5% recurrence rate (n = 35) with a mean recurrence timeline of 4.3 years (range: 6 months to 7 years). Similarly, Lionaki et al. [5] found a 7.7% recurrence rate (n = 26), only 1 lead to graft failure. 

Goral et al. [4] found recurrence in 15 of 50 patients (30%) with a mean time to biopsy of 51.4 ± 56 days (range: 6 – 184 days). Nyberg et al. [17] demonstrated a recurrence rate of 43.8% (7 out of 16 patients) with a mean follow-up time of 6 months (range: 0.5 – 11 years). Stone et al. [18] reported a recurrence rate of 9.2% (9 of 97 patients) an average of 3.1 years after transplantation. Contreras et al. [7] published the largest study of rLN involving 6,850 patients of which 167 (2.44%) experienced recurrence. Among these, 93% experienced graft failure and 86% experienced rejection. Recurrence was documented as early as the 1^st^ week and as late as 16 years post transplantation, with most of the events occurring during the first 10 years [7]. 

Despite variability in recurrence rates, most reported cases of rLN were generally mild and usually did not lead to allograft loss [12]. For example, of 15 cases of rLN reported by Goral et al. [4], 8 were class II, 4 were class III, and 3 were class V. Similarly, of 6 cases reported by Yu et al. [1], 3 were class I, 1 was class II, 1 was class IV, and 1 was class III + V. Norby et al. [2] found that 17 of 22 patients with rLN had subclinical lupus class I or II, and only 3 patients had class III or IV. Class IV suggests a worse prognosis; however, we demonstrate that class IV is still treatable. 

A positive C4d stain in the peritubular capillaries of a kidney allograft is suggestive of antibody interaction with the endothelium. Hence, it satisfies 2 of the 3 criteria required by Banff to diagnose antibody-mediated rejection. However, as our case illustrates, immune complex diseases such as lupus nephritis result in complement activation by the classic pathway producing a positive C4d stain. C4d deposition can be found in tubular basement membranes, glomeruli, peritubular capillaries, and arteries in patients with lupus nephritis. Our patient had electron-dense deposits abutting the basement membranes of the peritubular capillaries to explain the positive C4d stain. Indeed, there was no other evidence for antibody-mediated rejection at the time, nor has any appeared subsequently. There was no microvascular inflammation (g0 and ptc0) and no arteritis. There was no evidence of chronic antibody-mediated rejection, such as glomerular basement membrane reduplication or peritubular capillary basement membrane multilayering. Thus, when evaluating allograft biopsies of patients with underlying systemic lupus, a positive C4d stain is not an automatic indication of antibody-mediated rejection. 

## Conclusion 

Lupus nephritis can recur in the transplanted kidney far later than previously documented. We show that even at this late date, it is treatable, and our patient reinforces the need for long-term consideration of potential recurrence. Furthermore, in patients with ESKD from lupus nephritis that have received kidney transplants, a positive peritubular capillary stain for C4d may represent recurrent immune complex disease and not antibody-mediated rejection. 

## Authors’ contributions 

Jessica K Cobb: Conceptualization, data curation, project administration, visualization, preparing/editing manuscript. Paul Endres: Validation, preparing/editing manuscript/ drafting the work or revising it critically for important intellectual content. Prashamsa Shenoy: Validation, editing manuscript/drafting the work or revising it critically for important intellectual content. Edward J Filippone: Visualization, validation, supervisor, drafting the work or revising it critically for important intellectual content. Maitreyee Gupta: Visualization, validation, supervisor, drafting the work or revising it critically for important intellectual content. Rakesh Gulati: Conceptualization, supervisor, visualization, validation, drafting the work or revising it critically for important intellectual content. 

## Funding 

None. 

## Conflicts of interest 

None. 

**Table 1.** Laboratory data available for patient throughout time of lupus recurrence until present.

**Date**	**Cr (RR 0.76 – 1.27 mg/dL)**	**CRP RR 0 – 10 mg/L)**	**Anti-dsDNA (RR 0 – 9 IU/mL)**	**C3 (RR 82 – 185 mg/dL)**	**C4 (RR 15 – 53 mg/dL)**	**Prot:Cr ratio (RR 0 – 200 mg/g)**
Jul-13	1.3	–	–	–	–	–
Jul-15	1.43	–	–	–	–	–
May-20	1.51	17.2	16	73	5	500
Jun-20	1.51	6.1	17	80	8	425
Aug-20	1.48	6.6	14	89	12	143
Oct-20	1.4	9	14	92	10	250
Jan-21	1.22	4.6	12	71	7	128
May-21	1.4	19.2	11	84	10	–
Aug-21	1.69	–	11	75	8	128
Oct-21	1.27	4.6	13	71	7	–
Nov-22	1.4	8	12	90	5	161
Mar-23	1.26	–	10	83	5	–
Jan-24	1.26	8.6	–	102	13	124

RR = reference range; Cr = creatinine; CRP = C-reactive protein; Prot:Cr = protein-to-creatinine ratio.

**Table 2.** Breakdown of recurrence rate, time to recurrence, follow-up time, patients per class of lupus nephritis, and outcomes [1, 2, 3, 4, 7, 17, 18].

**Publication**	**Recurrence Rate**	**Time to recurrence**	**Mean follow-up**	**# Patients/class**
Yu et al. [1]	6/32 (26%)	5.1 years ± 4.9 years (1.8 months to 11.6 years)	10.2 ± 7.2 years	Class I - 3
	Outcomes: The graft and patient survival rates of recurrent lupus nephritis were not different from those of patients with other diagnoses. Although rLN was not uncommon, it did not appear to have a strong negative impact on long-term outcome in kidney transplant patients.			Class II - 1
				Class IV - 1
				Class III + V - 1
Norby et al. [2]	22/41 54%	8.3 years (3 to 18 years	N/A	Class I - 10
	Outcomes: Despite the high incidence of RLN, chronic allograft nephropathy was common in both patients with and without recurrence, and RLN did not significantly impact graft survival			Class II - 7
				Class III - 1
				Class IV - 1
				Class III + V - 1
				Class V - 2
Moroni et al. [3]	3/35 (8.5%)	4.3 years (6 months to 7 years)	91 ± 59 months	Class II - 1
	Outome: Patient one had acute cellular rejection 4 years after rLN iso IS noncompliance. Patient two had stable renal function for 8 years after rLN, then graft slowly deteriorated iso no change in IS. Patient three improved with ACEi.			Class III - 1
				Class V - 1
Goral et al. [4]	15/50 (30%)	51.4 days ± 56 days (6 days to 184 days)	6.8 ± 4.9 years	Class II - 8
	Outcomes: One patient lost the graft at 10.5 2/2 rLN (class II). The duration of dialysis before transplantation was not different between patients with RLN compared to patients without RLN (p = 0.40)			Class III - 4
				Class V - 3
Nyberg et al. [17]	7/16 (43.8%)	6 months to 11 years	5.6 years	N/A
	Outcomes: Upon increased IS therapy, renal and serological signs improved, but one graft was later lost due to rLN.			
Stone et al. [18]	9/97 (9.2%)	3.1 years (5 days to 9.3 years)	62.6 months	Class II - 4
	Outcomes: Loss of 3 allografts (1 class II, 2 class IV,1 class V). A higher percentage of patients with recurrence lost their graft 66.7% vs 48% (p = 0.18). 6 pts with recurrent LN ultimately lost their graft – with rLN clearly contributing to allograft loss in 4 of them.			Clas III - 1
				Class IV - 2
				Class V - 2
Contreras et al. [7]	167/6850 (2.44%)	7 days to 16 years	19 years	Class II - 28
	Outcomes: During follow-up, 156 (93.4%) recipients in the RLN group and 1517 (85.7%) recipients from the rejection group lost their allografts.			Class IV - 97
				Class V - 42

rLN = recurrent lupus nephritis; IS = immunosuppression; ACEi = Angiotensin-converting-enzyme inhibitors.
